# The Territorial Regimental Surgeon

**Published:** 1909-03-20

**Authors:** L. C. V. Hardwicke

**Affiliations:** Captain 1st London Division. Paddington Infirmary, Harrow Road, W.


*To the Editor of* The Hospital.

### THE TERRITORIAL REGIMENTAL SURGEON.

*To the Editor of* The Hospital.

Sir,—In the article on "The Appointment and Conditions of Service of the Territorial Regimental Surgeon" in last week's Hospital, one or two facts, or rather omissions, are rather misleading. The subjects in which an officer will be examined before promotion to the rank of captain are six in number—namely : (1) Drill; (2) war establishments; (3) organisations of field units; (4) Army regulations—discipline and duties of a company officer; (5) sanitation and other duties in camp, barracks, and on the line of march; (6) military law. The examination may be passed at any time after the officer has completed eighteen months' service, or before that time if in the interests of the Service. Also under the present conditions (the same held good under the Volunteer regulations) the £20 outfit allowance is not given until this examination has been passed, which means probably two years after joining.

These conditions are set out in a "Memorandum on the Medical Service of the Territorial Force," signed by the Director-General, Army Medical Service.

I am, etc.,
L. C. V. Hardwicke, M.B., R.A.M.C.T.,
Captain 1st London Division.

Paddington Infirmary, Harrow Road, W., March 8.

*** With reference to the above letter, the Editor of the R.A.M.C. Section of The Hospital writes as follows :—
Further consideration of the Territorial Regulations, which are not very explicitly drawn, makes it clear that our correspondent is right, and we are obliged for his drawing attention to the omission.

